# Identification of shared gene signatures and pathways for diagnosing osteoporosis with sarcopenia through integrated bioinformatics analysis and machine learning

**DOI:** 10.1186/s12891-024-07555-2

**Published:** 2024-06-03

**Authors:** Xiaoli Zhou, Lina Zhao, Zepei Zhang, Yang Chen, Guangdong Chen, Jun Miao, Xiaohui Li

**Affiliations:** 1 Department of Spine Surgery, Tianjin Hospital, Tianjin University, Tianjin, 300211 China; 2 Department of Toxicology, Tianjin Centers for Disease Control and Prevention, Tianjin, 300011 China; 3 Department of Orthopaedics, Cangzhou Central Hospital, Hebei, 061001 China; 4 The Third Central, Tianjin Key Laboratory of Extracorporeal Life Support for Critical Diseases, Clinical College of Tianjin Medical University, Nankai University Affinity the Third Central Hospital, Artificial Cell Engineering Technology Research Center, Tianjin Institute of Hepatobiliary Disease, Tianjin, 300170 China; 5 Department of Anaesthesiology, Tianjin Hospital, Tianjin, 300211 China; 6 Department of Joint Surgery, Tianjin Hospital, Tianjin University, Tianjin, 300211 China

**Keywords:** Sarcopenia, Osteoporosis, Hub genes, Machine learning, Bioinformatics

## Abstract

**Background:**

Prior studies have suggested a potential relationship between osteoporosis and sarcopenia, both of which can present symptoms of compromised mobility. Additionally, fractures among the elderly are often considered a common outcome of both conditions. There is a strong correlation between fractures in the elderly population, decreased muscle mass, weakened muscle strength, heightened risk of falls, and diminished bone density. This study aimed to pinpoint crucial diagnostic candidate genes for osteoporosis patients with concomitant sarcopenia.

**Methods:**

Two osteoporosis datasets and one sarcopenia dataset were obtained from the Gene Expression Omnibus (GEO). Differential expression genes (DEGs) and module genes were identified using Limma and Weighted Gene Co-expression Network Analysis (WGCNA), followed by functional enrichment analysis, construction of protein–protein interaction (PPI) networks, and application of a machine learning algorithm (least absolute shrinkage and selection operator (LASSO) regression) to determine candidate hub genes for diagnosing osteoporosis combined with sarcopenia. Receiver operating characteristic (ROC) curves and column line plots were generated.

**Results:**

The merged osteoporosis dataset comprised 2067 DEGs, with 424 module genes filtered in sarcopenia. The intersection of DEGs between osteoporosis and sarcopenia module genes consisted of 60 genes, primarily enriched in viral infection. Through construction of the PPI network, 30 node genes were filtered, and after machine learning, 7 candidate hub genes were selected for column line plot construction and diagnostic value assessment. Both the column line plots and all 7 candidate hub genes exhibited high diagnostic value (area under the curve ranging from 1.00 to 0.93).

**Conclusion:**

We identified 7 candidate hub genes (PDP1, ALS2CL, VLDLR, PLEKHA6, PPP1CB, MOSPD2, METTL9) and constructed column line plots for osteoporosis combined with sarcopenia. This study provides reference for potential peripheral blood diagnostic candidate genes for sarcopenia in osteoporosis patients.

## Introduction

Sarcopenia, a progressive skeletal muscle disorder, is linked to increased risks of falls, fractures, physical disability, and mortality[1]. While commonly observed in older adults, sarcopenia can also manifest earlier in life. Rooted in age-related muscle changes, it significantly reduces muscle strength and mass, contributing to heightened fall risks and impaired daily activities. This often leads to disability, loss of independence, and even death. The substantial impact of sarcopenia on morbidity, mortality, and healthcare expenditure has spurred significant research and policy discussions, underscoring its critical importance. Decreased muscle strength, crucial for mobility, significantly increases fall prevalence among the elderly. This condition correlates closely with self-reported physical disability, transcending factors such as ethnicity, age, morbidity, obesity, income, or health behaviors[2]. Age-related decline in muscle strength not only diminishes functional capacity but also exacerbates disability, mortality, and other adverse health outcomes[3]. With the aging population, sarcopenia-related morbidity is expected to pose a significant healthcare challenge. Management strategies for sarcopenia include non-pharmacological interventions like resistance exercise and proper nutrition, notably protein intake and vitamin D supplementation. Resistance exercise, in particular, is a standard non-pharmacological treatment, supported by substantial evidence. While various pharmacological agents have shown efficacy, future research should focus on elucidating biological pathways, refining diagnostics, and developing superior treatment methods[4].

Osteoporosis and related fractures are prevalent among older adults, posing substantial morbidity and mortality risks. Bisphosphonates are the primary therapy, with additional options like denosumab, teriparatide, and selective estrogen receptor modulators available. Early identification and intervention for osteoporosis are crucial for mitigating its effects[5]. The coexistence of osteoporosis and sarcopenia, termed 'osteosarcopenia'[5], poses a dual challenge. Interactions between muscles and bones at various levels may contribute to osteosarcopenia's pathophysiology [6]. Understanding shared genes between these systems could offer novel treatment insights.

In this study, instrumental variables at the genome-wide significance level were utilized to assess the bi-directional causality between sarcopenia and osteoporosis. The results suggest a potential mutual influence between the two conditions. The study also highlights the frequency of osteosarcopenia and its association with increased fracture risks. Standardized classification of sarcopenia is crucial for accurately assessing its relationship and consequences [7]. Muscles and bones, originating from mesodermal and ectodermal mesenchymal stem cells, share close anatomical proximity, facilitating mechanical and chemical signal exchange. Identifying shared crosstalk genes could offer novel prevention and treatment avenues [8]. After identifying shared genetic markers, validate the functionality of these genes using cellular or animal models, and determine their potential mechanisms in disease progression. Develop molecular diagnostic tests based on blood or tissue samples for screening the risk of muscle atrophy and osteoporosis. Develop gene therapy approaches or novel drugs targeting the expression or functionality of these genes.

## Material and methods

### Data collection

**Fig. 1:**
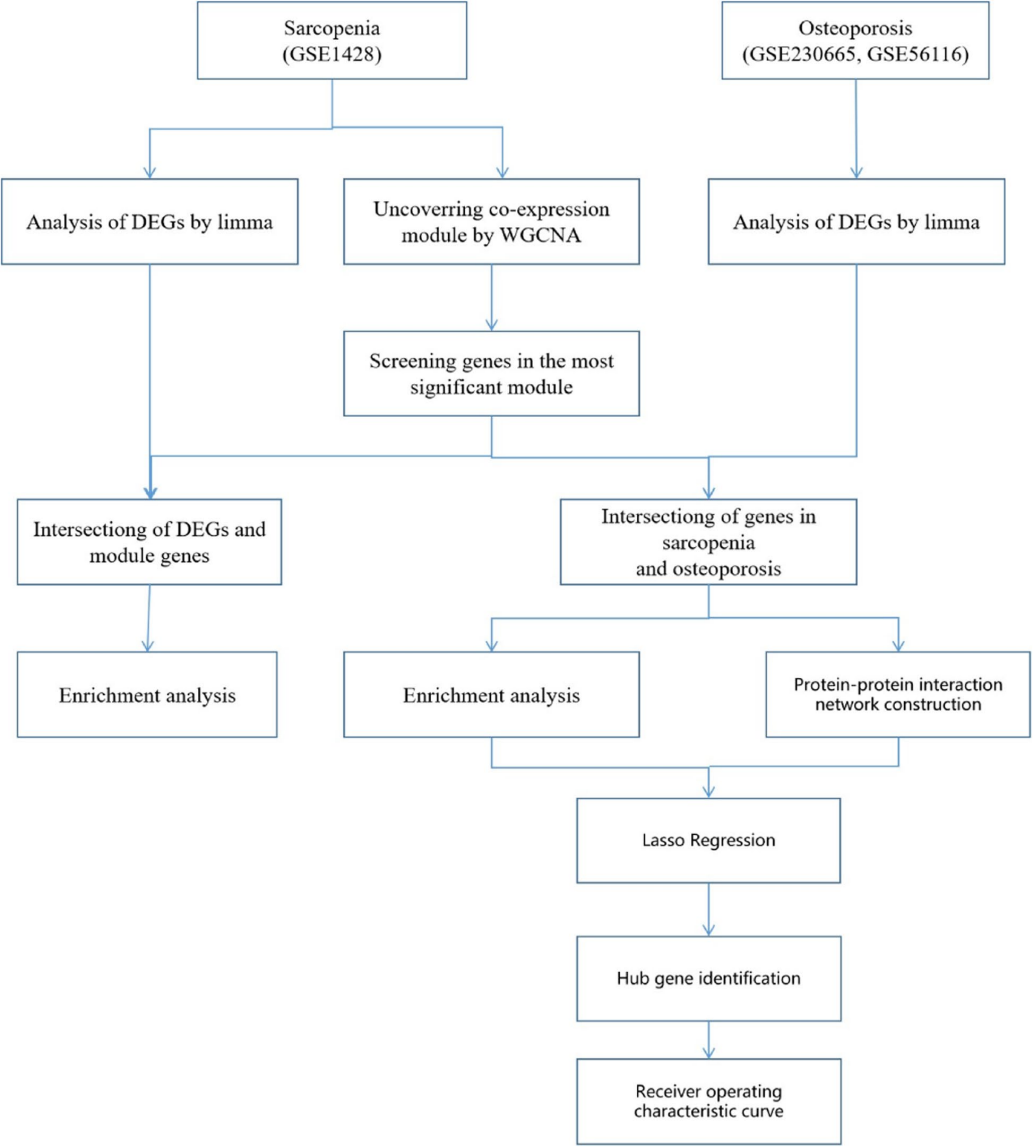
Workflow of the whole study

The datasets GSE1428 and GSE230665, as well as GSE56116, were curated from the GEO database (https://www.ncbi.nlm.nih.gov/geo/) [9]. For the microarray analysis of the GSE1428 [10] dataset, the GPL96 platform (Affymetrix Human Genome U133A Array) was utilized. GSE230665 [11] employed the GPL10332 platform (Agilent-026652 Whole Human Genome Microarray 4 × 44 K v2, Feature Number version). GSE56116 [11] utilized the GPL4133 platform (Agilent-014850 Whole Human Genome Microarray 4 × 44 K G4112F, Feature Number version). The GSE1428 dataset presents transcriptional responses related to sarcopenia, as provided by Giresi et al. On the other hand, the datasets GSE230665 and GSE56116, focusing on osteoporosis, were contributed by Ge, Li, and their respective collaborators. The GSE1428 dataset showcases the transcriptional responses associated with sarcopenia, while GSE230665 and GSE56116 pertain to datasets on osteoporosis (Fig. 1).

### Identification of DEGs between sarcopenia and osteoporosis

For the two original osteoporosis datasets, empirical Bayes methods [12] were applied to eliminate batch effects. The merged osteoporosis dataset and the sarcopenia dataset underwent the extraction of expression matrices, with the exclusion of genes and samples featuring missing values exceeding 50%. Subsequently, missing values were imputed using the "impute.knn" function from the R package "impute," setting the Number of neighbors to 10 for data completion. Furthermore, a log2 transformation was applied to the data. In cases where multiple probes identified the same gene, the average expression was calculated. Finally, utilizing the Limma package, criteria of |log2 Fold change (FC)|> 1.5 and *P* < 0.05 were set as the standards for identifying DEGs. The definition of this threshold range refers to the study by Liu et al. [13].

### Weighted gene co-expression network analysis and module gene selection

Exploring gene–gene correlations using systems biology strategy WGCNA [14]. Based on gene expression profiles, we computed the median absolute deviation for each gene and removed the bottom 50% of genes with the smallest median absolute deviation. We utilized the R software package WGCNA's goodSamplesGenes method to eliminate outlier genes and samples. Subsequently, we employed WGCNA to construct a scale-free co-expression network. Initially, Pearson's correlation matrices and the average linkage method were applied to all pairwise genes. Then, a weighted adjacency matrix was created using a power function A_mn =|C_mn|^β (where C_mn represents the Pearson's correlation between gene_m and gene_n, and A_mn denotes the adjacency between gene m and gene n). The parameter β was chosen to be 12 for soft-thresholding, emphasizing strong correlations and penalizing weak ones. The adjacency matrix was transformed into a topological overlap matrix, measuring the network connectivity of a gene, defined as the sum of its adjacencies with all other genes for network gene ration, and the corresponding dissimilarity (1-TOM) was computed. To classify genes with similar expression profiles into gene modules, average linkage hierarchical clustering was performed based on the TOM-based dissimilarity measure, with a minimum module size of 100 for the gene dendrogram. We set the sensitivity to 3. To further analyze the modules, we calculated the dissimilarity of module eigen genes, selected a cut line for the module dendrogram, and merged some modules. Additionally, we merged modules with a distance less than 0.25, resulting in the identification of 2 co-expression modules.

### Functional enrichment analysis

The Gene Ontology (GO) [15] system provides structured and computable information about the functions of genes and gene products. The Kyoto Encyclopedia of Genes and Genomes (KEGG) [16] is a widely used database for gene system research. Using gene annotations from the R package org.Hs.eg.db (version 3.1.0), and obtaining the latest KEGG Pathway gene annotations from the KEGG rest API (https://www.kegg.jp/kegg/rest/keggapi.html) as background, genes were mapped to the background set. Functional enrichment analysis was performed using the R package clusterProfiler (version 3.14.3) to obtain results of gene set enrichment. The minimum gene set was set to 5, and the maximum gene set was set to 5000. A P value of < 0.05 and a false discovery rate of < 0.1 were considered statistically significant. Two rounds of GO and KEGG analyses were conducted based on the intersection of DEGs in sarcopenia and the most significant module genes, as well as the intersection of DEGs in osteoporosis and the most significant module genes in sarcopenia.

### Construction of protein–protein interaction network

To explore the interactions between protein-coding genes, we utilized the String database [17] (version 11.5; www.string-db.org), with a minimum interaction score set to 0.400. The obtained network from String was further modified using Cytoscape software. All interacting genes within the protein–protein interaction (PPI) network were selected for subsequent analysis.

### Machine learning

To further screen candidate genes for diagnosing sarcopenia and osteoporosis, a machine learning algorithm was employed. LASSO [18] (Least Absolute Shrinkage and Selection Operator) is a regression method used for variable selection to improve prediction accuracy. It is also a regularization technique that enhances the predictive accuracy and interpretability of statistical models. Utilizing the R package glmnet, gene expression data was integrated, and regression analysis was performed using the lasso-cox method. A threefold cross-validation was set up to obtain the optimal model. By dividing the dataset into 3 parts and rotating 2 of them for model training while keeping 1 for testing, we iterated through this process. In each iteration, we evaluated the performance of the model under different λ values, primarily by observing the prediction errors of the model. Finally, we selected the λ value that minimized the cross-validation error as the optimal λ. The Lambda value was set to 0.0639847346226388. The genes obtained from this analysis were identified as candidate hub genes for diagnosing sarcopenia and osteoporosis.

### Construction of column line plots and ROC curve evaluation

Construction of column line plots holds certain value in diagnosing clinical sarcopenia and osteoporosis. Using the candidate genes, column line plots were constructed using the R package pROC (version 1.17.0.1). The "Score" represents the score of the candidate genes, while "Total Score" represents the sum of scores for all the aforementioned genes. ROC curves were established to evaluate the diagnostic value of the candidate genes and column line plots for sarcopenia and osteoporosis.

## Results

### Identification of DEGs

**Fig. 2:**
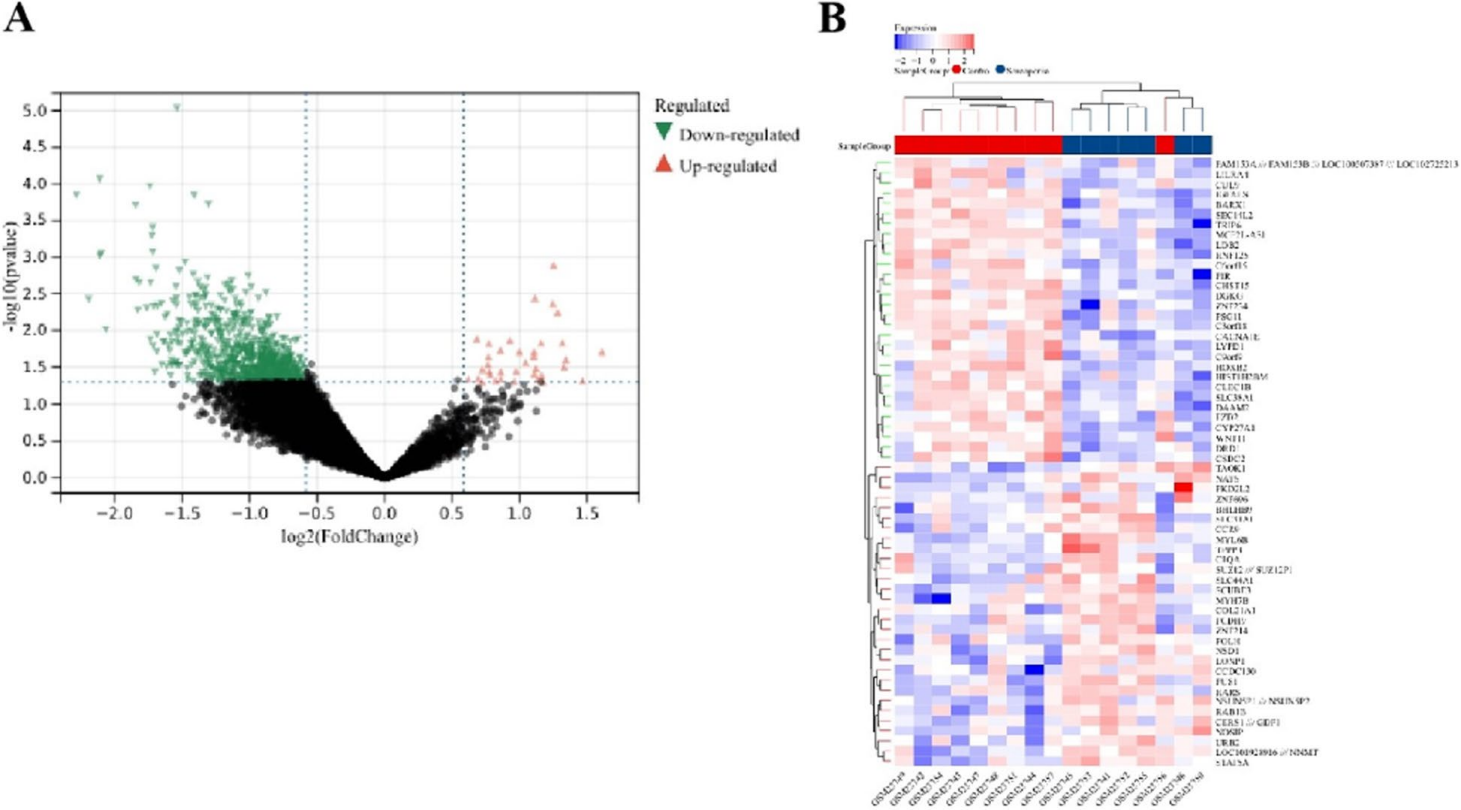
Heatmap and volcano plot for the DEGs identified from the sarcopenia dataset

**Fig. 3:**
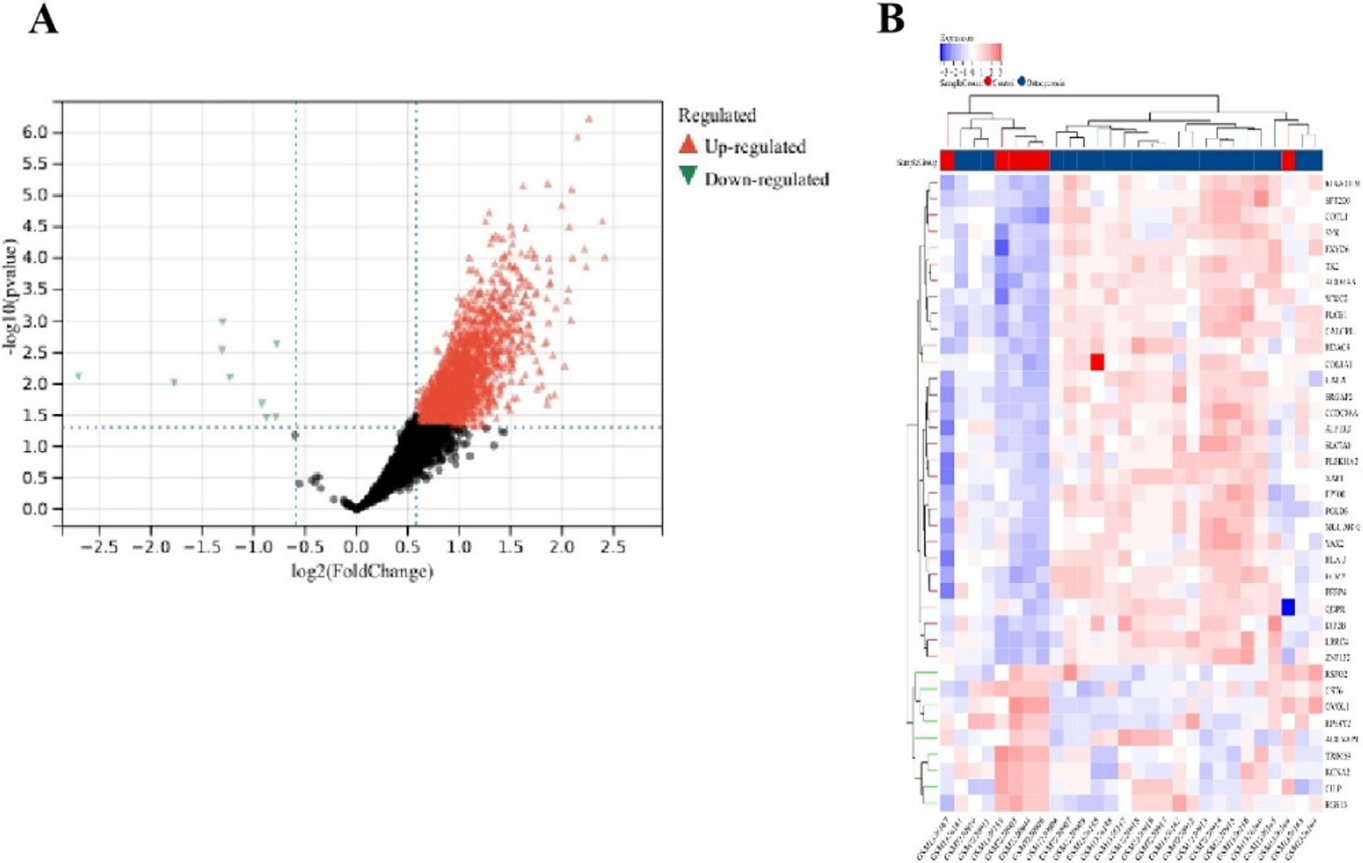
Heatmap and volcano plot for the DEGs identified from the osteoporosis dataset

Identification of DEGs using Limma method revealed a total of 821 DEGs (337 upregulated, 484 downregulated) in the sarcopenia dataset. The heatmap and volcano plot of sarcopenia DEGs are shown in Fig. 2A-B. In the combined osteoporosis dataset, a total of 2067 DEGs were identified, with 2059 upregulated and 9 downregulated genes. The heatmap and volcano plot of osteoporosis DEGs are illustrated in Fig. 3A-B.

### WGCNA analysis and identification of key modules

**Fig. 4:**
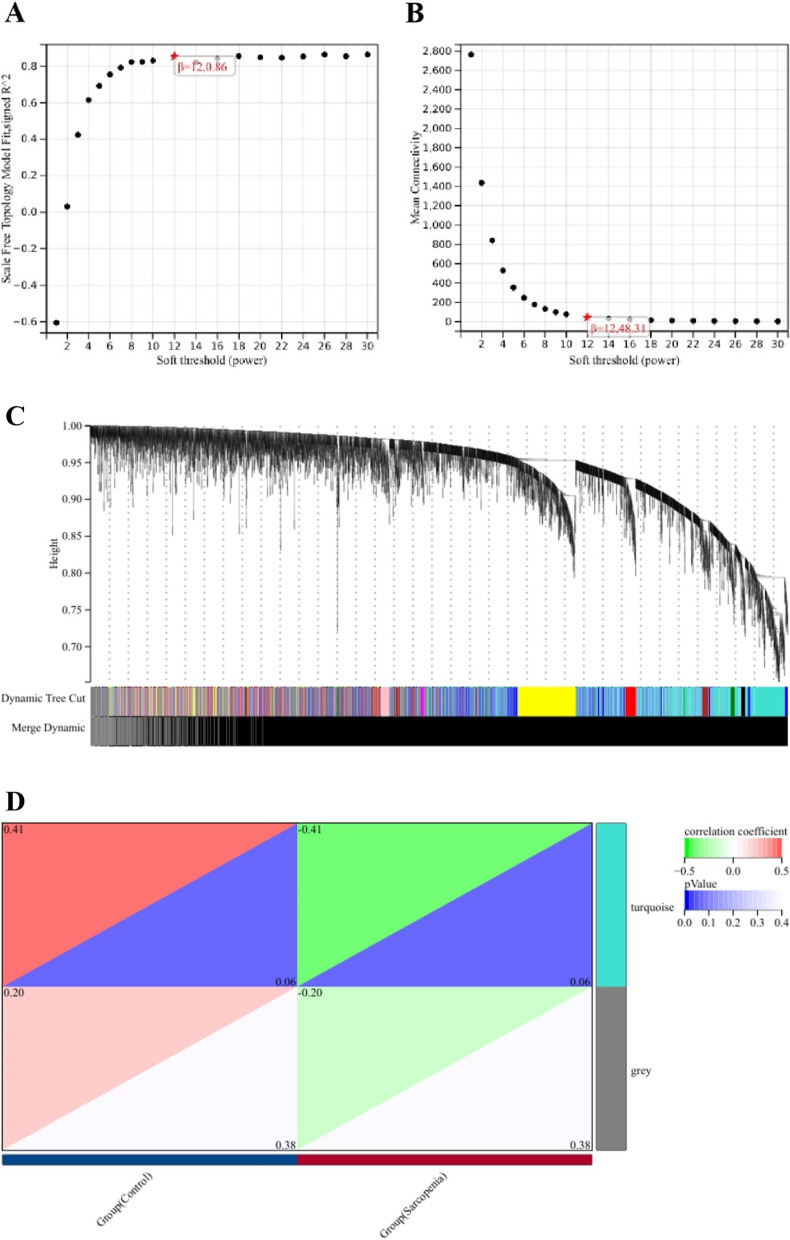
WGCNA of DEGs.** A-B **Estimation of the soft thresholding value for a scale-free co-expression network. **C** Cluster dendrogram of all DEGs. **D** Heatmap showing the correlation between modules and sarcopenia. The turquoise module is found to be significantly correlated with sarcopenia. The numbers in the top and bottom brackets represent the correlation coefficient and *p*-value, respectively.

We selected β = 12 (scale-free R^2^ = 0.86) as the "soft" threshold based on scale independence and average connectivity (Fig. 4A-B). The dendrogram (Fig. 4C) depicts the clustering of sarcopenia and control samples. Based on this, two gene co-expression modules were generated, as indicated by different colors in Fig. 4D. Among them, the turquoise module (424 genes) exhibited the highest correlation with sarcopenia (correlation coefficient = -0.41, *P* = 0.06) and was considered the key module for subsequent analysis.

### Functional enrichment analysis of sarcopenia

**Fig. 5:**
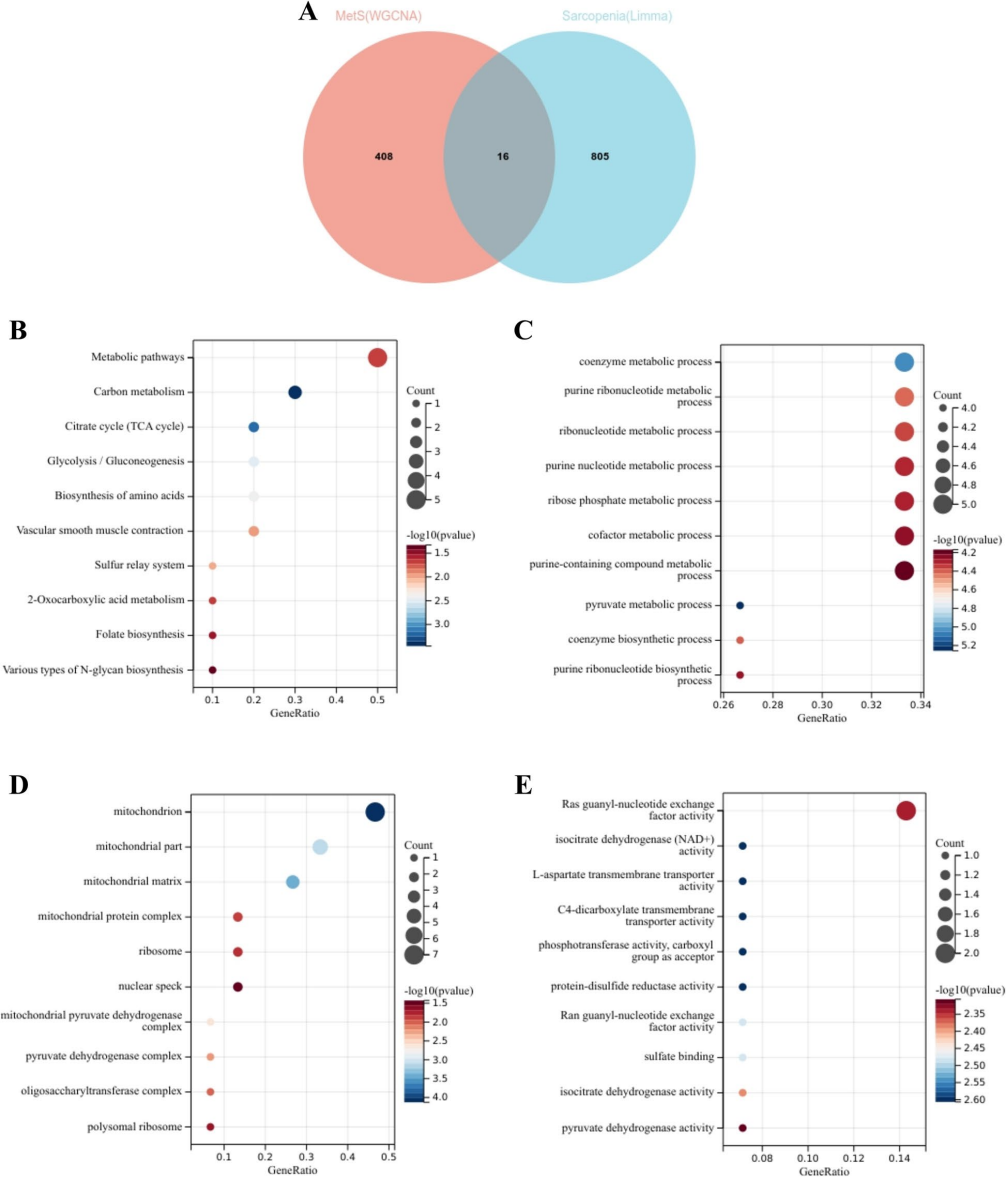
Enrichment analysis of the intersection of genes in sarcopenia.** A** Venn diagram shows that 16 genes are identified from the intersection of DEGs via Limma and green module genes via WGCNA. **B** KEGG pathway analysis of the intersection of genes. Different colors represent various significant pathways and related enriched genes. **C-E** GO analysis of the intersection of genes, including biological process, cellular component, and molecular function, respectively. The y-axis represents different GO terms, the x-axis represents gene ratio enriched in relative GO terms, the circle size refers to gene numbers, and the color represents *p* value.

To assess whether the dataset GSE1428 reliably reflects the pathogenesis of sarcopenia, we further conducted functional enrichment analysis based on the intersection of Limma and WGCNA module genes. The intersection of 424 DEGs from the turquoise module with 821 genes yielded 16 common genes (Fig. 5A). KEGG analysis revealed that common genes were primarily enriched in "Metabolic pathways" and "Carbon metabolism" (Fig. 5B). GO analysis indicated that common genes were predominantly enriched in biological process (BP) terms, including "coenzyme metabolic process" and "purine ribonucleotide metabolic process" (Fig. 5C). Regarding cellular component (CC) ontology, CGs were mainly located in "mitochondrion," "mitochondrial part," and "mitochondrial matrix" (Fig. 5D). Molecular function (MF) analysis showed that "Ras guanyl-nucleotide exchange factor activity," "isocitrate dehydrogenase (NAD +) activity," and "L-aspartate transmembrane transporter activity" were the most significant terms within common genes (Fig. 5E).

### Enrichment analysis and node gene identification for osteoporosis and sarcopenia based on PPI networks

**Fig. 6:**
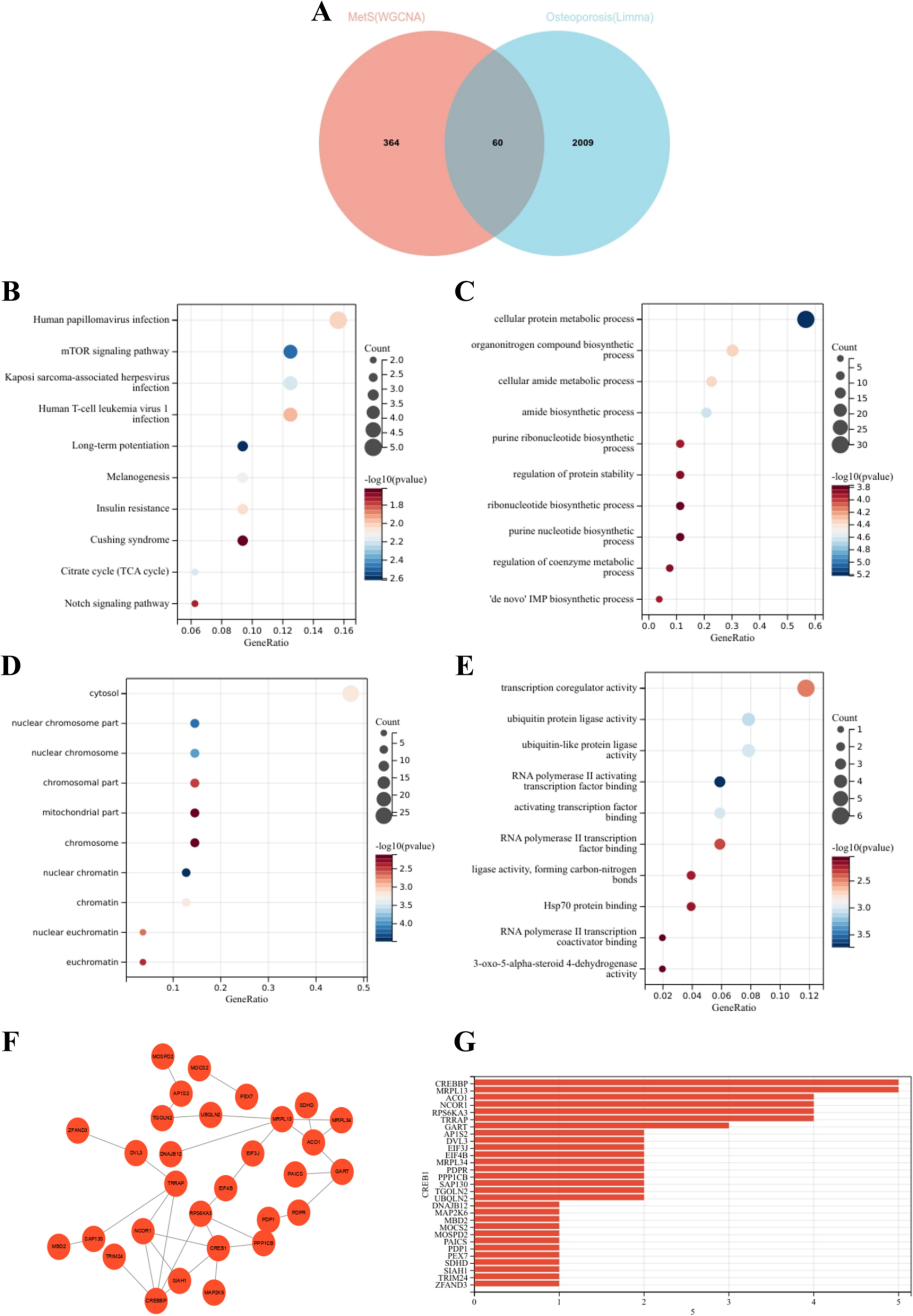
Enrichment analysis of common genes from osteoporosis with sarcopenia and the identification of node genes from PPI network. **A** Venn diagram shows that 60 common genes are identified from the intersection of genes in osteoporosis using Limma and sarcopenia using WGCNA. **B** KEGG analysis of 60 common genes. **C-E** GO analysis (biological process, cellular component, and molecular function) of 60 common genes. **F** PPI network reveals that 30 genes interact with each other. **G** The column shows the gene nodes of 26 genes in PPI network.

To further explore whether key genes associated with sarcopenia are also related to the pathogenesis of osteoporosis, we visualized the intersection of DEGs in osteoporosis and module genes in sarcopenia through a Venn diagram, identifying 60 genes (Fig. 6A). KEGG enrichment analysis revealed that these 60 genes were mainly enriched in "Human papillomavirus infection," "mTOR signaling pathway," and "Kaposi sarcoma-associated herpesvirus infection" (Fig. 6B). GO analysis showed that these genes were enriched in "Kaposi sarcoma-associated herpesvirus infection," "organonitrogen compound biosynthetic process," and "cellular amide metabolic process" in BP; "cytosol," "cytosol," and "nuclear chromosome" in CC; and "transcription coregulator activity," "transcription coregulator activity," and "ubiquitin-like protein ligase activity" in MF (Fig. 6C-E).

After confirming the filtered genes, we constructed a PPI network to identify interacting node genes for subsequent machine learning filtering. Figure 6F displays the PPI network, where 30 genes can interact with each other. These genes are sorted by node degree in Fig. 6G

### Identifying candidate hub genes through machine learning

**Fig. 7:**
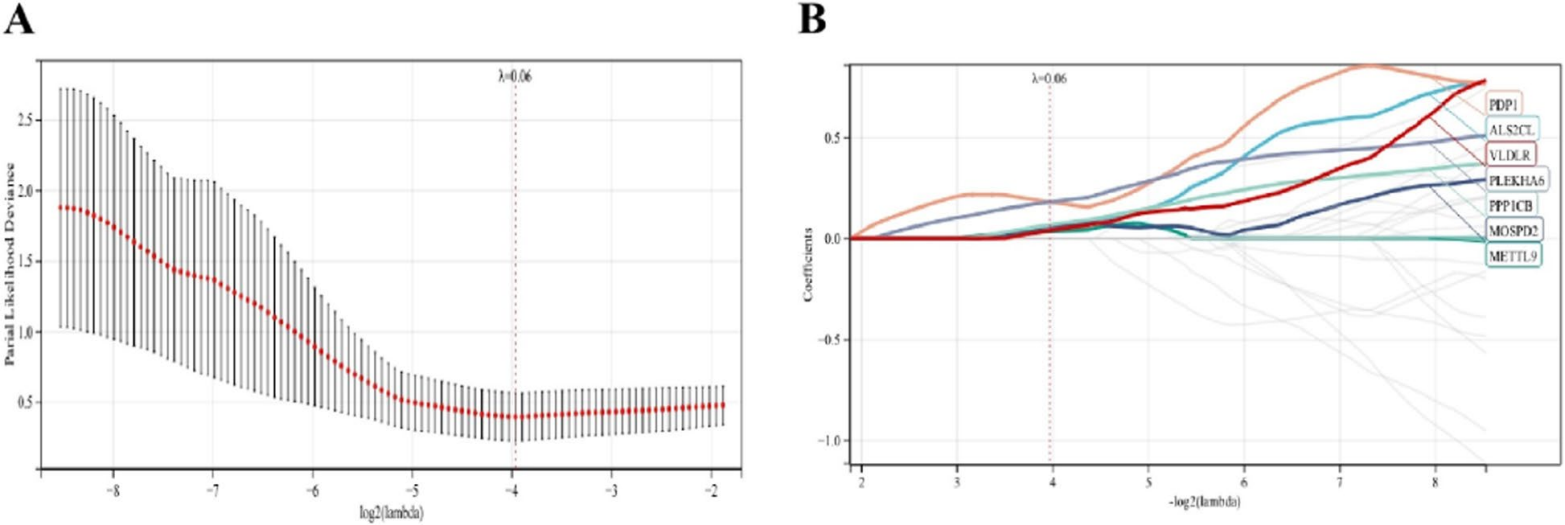
Machine learning in screening candidate diagnostic biomarkers for osteoporosis with sarcopenia. **A-B** Biomarkers screening in the Lasso model. The number of genes (*n*=7) corresponding to the lowest point of the curve is the most suitable for osteoporosis with sarcopenia diagnosis.

The LASSO regression machine learning algorithm was applied to select candidate genes for column line plot construction and diagnostic value assessment. From Fig. 7A-B, it can be observed that the LASSO regression algorithm identified 7 potential candidate biomarkers for final validation.

### Diagnostic Value Evaluation

**Fig. 8:**
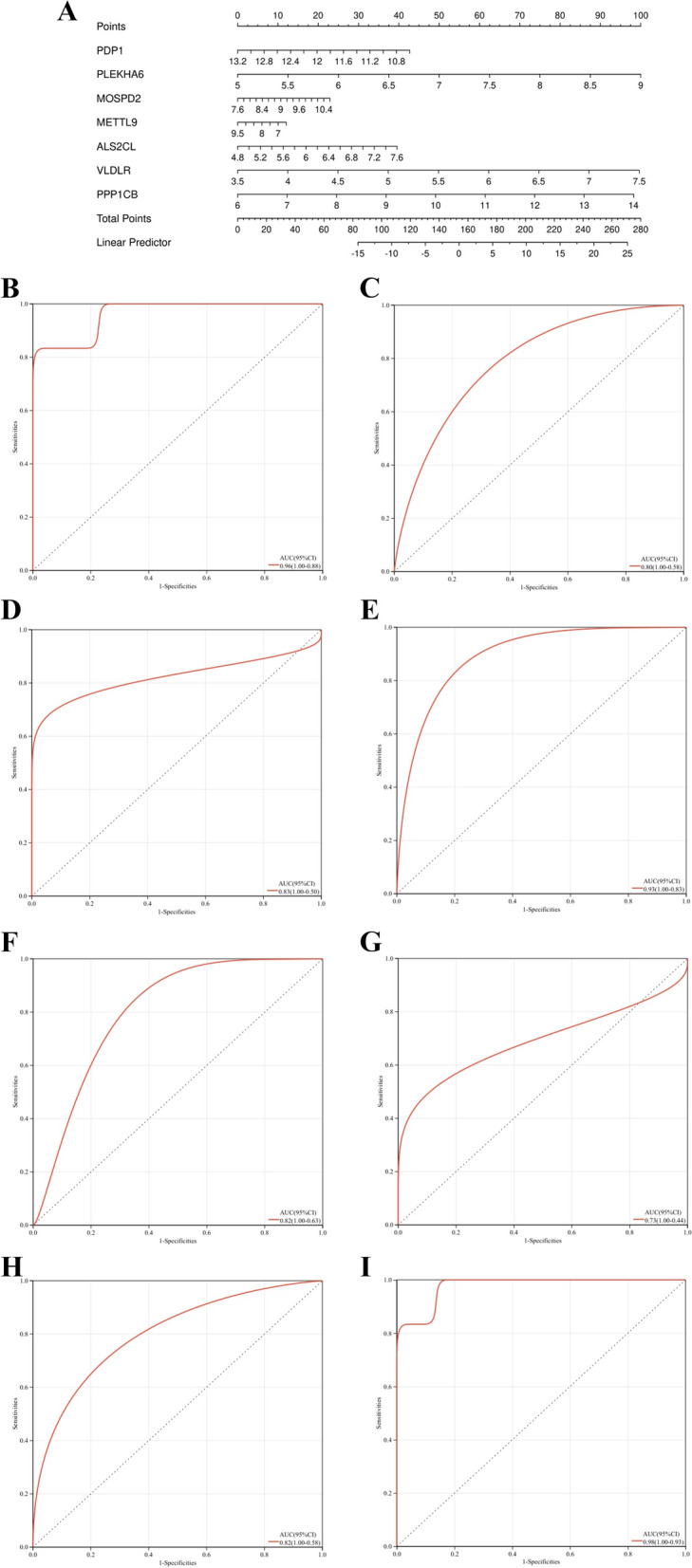
8 Nomogram construction and the diagnostic value evaluation. **A** The visible nomogram for diagnosing osteoporosis with sarcopenia. **B-I** The ROC curve of each candidate gene (PDP1, ALS2CL, VLDLR, PLEKHA6, PPP1CB, MOSPD2, METTL9) and nomogram show the significant steoporosis with sarcopenia diagnostic value.

Based on the 7 candidate hub genes, a column line plot was constructed (Fig. 8A), and ROC curves were established to evaluate the diagnostic specificity and sensitivity of each gene and the column line plot. The area under the curve (AUC) and its 95% confidence interval (CI) were calculated for each item. The results are as follows: PDP1 (AUC 0.96, CI 1.00 ~ 0.88), ALS2CL (AUC 0.80, CI 1.00 ~ 0.58), VLDLR (AUC 0.83, CI 1.00 ~ 0.5), PLEKHA6 (AUC 0.93, CI 1.00 ~ 0.83), PPP1CB (AUC 0.82, CI 1.00 ~ 0.63), MOSPD2 (AUC 0.73, CI 1.00 ~ 0.44), METTL9 (AUC 0.82, CI 1.00 ~ 0.58), and the column line plot (AUC 0.98, CI 1.00 ~ 0.93) (Fig. 8B-I). All candidate genes exhibited high diagnostic value for sarcopenia combined with osteoporosis, with the column line plot demonstrating the highest diagnostic value.

## Discussion

Osteosarcopenia, characterized by the coexistence of osteopenia/osteoporosis and sarcopenia, has emerged as a significant health concern, imposing a substantial global health burden. According to the World Health Organization, osteopenia and osteoporosis are defined by T scores equal to or less than − 1 and − 2.5 standard deviations, respectively, below the peak bone mass of a young, healthy cohort or in the presence of a minimal-trauma fracture. This skeletal condition results in the deterioration of bone microarchitecture and compromises bone strength [19]. Conversely, sarcopenia is identified by cut-off values indicating low muscle mass, strength, and/or functional capacity [20]. Both osteosarcopenia and sarcopenia share common risk factors [21] and exhibit strong associations with frailty, falls, fractures, hospitalizations, and mortality [21–23], contributing to a significant increase in healthcare expenditure. The coexistence of these conditions underscores the intricate interplay between skeletal and muscular health and emphasizes the need for comprehensive approaches to address their shared impact on overall well-being.

In this study, we utilized a series of integrated bioinformatics analyses and machine learning methods to construct a nomogram and evaluate the diagnostic value of osteoporosis in sarcopenia patients. A notable finding is the identification of 7 key candidate genes (PDP1, ALS2CL, VLDLR, PLEKHA6, PPP1CB, MOSPD2, and METTL9), and the development of a nomogram for diagnosing osteoporosis in sarcopenia patients.

The sarcopenia patient dataset used in this study all comes from peripheral blood samples. Therefore, we only need to collect peripheral blood samples from sarcopenia patients and evaluate the expression of the 7 identified immune-related genes to infer the probability of sarcopenia patients developing osteoporosis. This is an efficient and practical clinical approach. The use of peripheral blood testing in diagnosing various diseases is also widely accepted. Furthermore, although we confirmed that gene expression levels can serve as independent diagnostic markers, we plan to develop a more comprehensive diagnostic model by transforming them into scores and considering all 7 markers. The expression of each gene is quantified and converted into a score, with an increase in score indicating a higher linear prediction factor. When the linear prediction factor is high, we can conduct early monitoring and intervention in sarcopenia patients, which is more valuable for implementing osteoporosis diagnosis in sarcopenia. Using machine learning to identify pseudo-gene features for bone sarcoma prognosis, these four pseudo-gene features not only serve as promising indicators for predicting prognosis and survival rates but also represent potential markers for monitoring treatment regimens [24].

PDP1encodes a protein that is one of the three components (E1, E2, and E3) of the large pyruvate dehydrogenase complex. PDP1 plays a crucial role in protein phosphorylation and has been implicated in various diseases [25, 26]. Research has shown that miR-18a-3p improves cartilage matrix remodeling and suppresses inflammation in osteoarthritis by targeting PDP1 [27]. In pancreatic cancer, PDP1 promotes cancer proliferation and invasion by regulating the MAPK/mTOR signaling pathway [28]. Additionally, PDP1 is associated with osteosarcoma progression, patient prognosis, and chemosensitivity, making it a potential biomarker for osteosarcoma [29]. Given its role in multiple diseases, PDP1 is considered a potential diagnostic target for osteoporosis in sarcopenic patients. ALS2CL encodes a 108-kD protein with specific but relatively weak Rab5-GEF activity and strong Rab5-binding properties. Co-expression of ALS2CL and Rab5A in HeLa cells results in a unique tubulation phenotype of endosome compartments, indicating ALS2CL's involvement in modulating Rab5-mediated endosome dynamics [30]. VLDLR, or Very Low Density Lipoprotein Receptor, belongs to the low-density lipoprotein receptor family, with high expression levels in the brain, heart, skeletal muscle, and adipose tissue, while its expression in the liver is very low under physiological conditions. It plays a crucial role in controlling serum triglycerides and the development of non-alcoholic fatty liver disease. Previous studies have demonstrated the involvement of VLDLR in regulating the onset of various diseases. Research has found that homozygous loss-of-function mutations in VLDLR lead to dysequilibrium syndrome, a non-progressive cerebellar ataxia syndrome associated with intellectual disability [31]. PLEKHA6, along with other members of the WW-PLEKHA family, plays a role in the trafficking and retention of transmembrane proteins, including nectins, Tspan33, and the copper pump ATP7A, at cell–cell junctions and lateral membranes. Its C-terminal region and coiled-coil region promote its localization at adherens junctions of epithelial cells. This suggests that PLEKHA6 is involved in maintaining cell–cell adhesion and potentially regulates signaling pathways associated with adherens junctions [32]. PPP1CB, located on chromosome 2p23.2, encodes a subunit of PPP1 involved in various cellular functions, including glycogen metabolism, cell division, and muscle contraction [33–36]. Recent studies have identified PPP1CB as the myosin light chain phosphatase responsible for Ca2 + -transient rise and enhanced cell shortening in cardiomyocytes [37]. MOSPD2, a member of the VAP family, facilitates contact between the endoplasmic reticulum and various cellular organelles [38]. Unlike other VAP family members, MOSPD2 contains an additional cytoplasmic domain called CRAL-TRIO, which may be involved in lipid transport [39]. Research suggests that MOSPD2 is a key regulator of inflammation-driven monocyte migration and a potential therapeutic target for CNS inflammatory diseases [40]. METTL9, a methyltransferase, plays a crucial role in histone methylation and is implicated as an oncogene in various cancers [41, 42]. Targeting METTL9 significantly inhibits the growth of hepatocellular carcinoma patient-derived xenografts [43] and correlates with increased metastatic activity in human gastric cancer [44].

### Limitation

In summary, this study identified candidate hub genes for diagnosing osteoporosis combined with sarcopenia using integrated bioinformatics and machine learning approaches. However, limitations include reliance on publicly available datasets, potential selection bias in gene identification, limited generalizability to diverse populations, cross-sectional data analysis, and the need for further experimental validation to elucidate the functional mechanisms of the identified genes. These findings provide a foundation for potential peripheral blood diagnostic markers but require additional validation and clinical translation for practical application in healthcare settings. Examine the expression levels of these candidate genes in clinical samples using techniques such as real-time quantitative PCR or immunohistochemistry. Compare the expression differences of these genes between osteoporosis patients and healthy controls, as well as between muscle atrophy patients and healthy controls. Use statistical methods to determine the presence of significant correlations and evaluate the feasibility of these genes as potential biomarkers. Investigate the functions of these genes through cellular or animal models, especially their effects on bone and muscle tissue. For example, the impact of these genes on bone and muscle development and maintenance can be studied through gene knockout or overexpression. Conduct clinical cohort studies to track the disease progression and treatment response of patients with osteoporosis accompanied by muscle atrophy, and assess the potential of these candidate genes as predictive or prognostic markers. Carry out drug intervention trials to evaluate the efficacy of drug treatments targeting these genes for osteoporosis with muscle atrophy.

## Conclusion

Our study systematically identified seven candidate hub genes (PDP1, ALS2CL, VLDLR, PLEKHA6, PPP1CB, MOSPD2, and METTL9) through a combination of various bioinformatics analyses and machine learning algorithms, and provided a nomogram for diagnosing sarcopenia associated with osteoporosis. The research offers reference for potential peripheral blood diagnostic candidate genes for sarcopenia related to osteoporosis.

## Data Availability

The datasets analysed during the current study are available in Gene Expression Omnibus (GEO) database (http://www.ncbi.nlm.nih.gov/geo/):GSE1428,GSE230665,GSE56116.

## Abbreviations

AUC: Area under the curve
BP: Biological processes
CC: Cellular components
CI: Confidence interval
DEGs: Differentially expressed genes
GEO: Gene Expression Omnibus
GO: Gene Ontology
KEGG: Kyoto Encyclopedia of Genes and Genomes
LASSO: Least Absolute Shrinkage and Selection Operator
MF: Molecular functions
PDP1: Pyruvate dehydrogenase phosphatase catalytic subunit 1
PPI: Protein-protein interaction
ROC: Receiver operating characteristic
TOM: Topological overlap matrix
WGCNA: Weighted Gene Co-expression Network Analysis
